# Changes in DOM Quality Determine Prokaryotic Activities and Extracellular Release in the NW Mediterranean Sea: An Experimental Approach

**DOI:** 10.1111/1758-2229.70288

**Published:** 2026-05-07

**Authors:** Eva Ortega‐Retuerta, Nawal Bouchachi, Rebeca Campos, Olivier Crispi, Barbara Marie, Charles‐Hubert Paulin, Karine Escoubeyrou, Jonathan Colombet, Telesphore Sime‐Ngando, Anabel Von Jackowski

**Affiliations:** ^1^ Laboratoire d'Océanographie Microbienne UMR7621 CNRS/Sorbonne Université Banyuls‐sur‐Mer France; ^2^ Oceanographic Observatory of Banyuls‐sur‐Mer (OOB) Banyuls‐sur‐Mer France; ^3^ CNRS Laboratoire Microorganismes: Génome Environnement UMR 6023 Aubière France

## Abstract

Marine dissolved organic matter (DOM) has varying degrees of bioavailability from labile to refractory fractions. DOM bioavailability affects its prokaryotic uptake and release, which determines the carbon sequestration mechanism through the microbial carbon pump (MCP). Here, we tested how changes in DOM bioavailability influence prokaryotic communities and DOM transformation during an incubation experiment in the NW Mediterranean Sea. DOM was extracted at 5 m on 31 May and 26 August 2021, added to mesopelagic prokaryotic communities, and incubated for 34 days. Our results suggest that the growth of different prokaryotic communities, linked to the use of alkaline phosphatase and higher viral abundances, results in the release of prokaryotic‐derived DOM, which we traced using dissolved hydrolysable amino acids and the accumulation of humic‐like fluorescent DOM. The accumulation coincided with the growth of particular prokaryotic members, such as Campylobacterales and Enterobacterales, during the active growth phase, and Flavobacterales of the genera *Tenacibaculum*, Pedosphaerales and Chitinophagales at the end of the incubation. The parallel analysis of DOM and the prokaryotic community links DOM transformations with a temporal succession of niches in the free‐living prokaryotic community, identifying the main actors of the MCP of the Mediterranean Sea.

## Graphical abstract

Changes in DOM composition influence prokaryotic species growth in the Mediterranean Sea, shaping their metabolic responses and organic matter processing. In our study, higher DOP consumption and alkaline phosphatase activity led to the accumulation of recalcitrant DOM, highlighting how microbial community shifts drive distinct pathways in carbon transformation and sequestration.

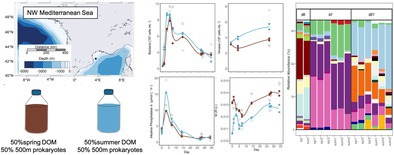

## 1 Introduction

Marine dissolved organic matter (DOM) is one of the highest pools of reduced carbon on Earth and plays fundamental roles in carbon sequestration through the biological and microbial carbon pumps (MCP; Jiao et al. 2010; Boyd et al. 2019). DOM is a diverse and dynamic marine carbon reservoir composed of thousands of different compounds that make its' complete chemical characterisation difficult (Hansell 2013; Dittmar 2015). Therefore, DOM is frequently classified using different criteria, such as reactivity or optical properties. According to dissolved organic carbon (DOC), the main component of DOM, the reactivity spectrum can be classified from labile to recalcitrant: labile DOC (LDOC) with a turnover of hours to days, semi‐labile DOC (SLDOC) with a turnover of weeks to months, while semi‐refractory DOC (SRDOC), refractory DOC (RDOC) and ultra‐refractory DOC (UDOC) have residence times of years to millennia (Hansell 2013). LDOC is typically only measured during phytoplankton blooms, while SLDOC and SRDOC fractions accumulate in the surface ocean depending on the season (Carlson et al. 1994; Hansell 2013). DOC accumulated in the deep ocean has been widely assumed to be refractory, but this is currently under debate since photochemical reactions remove RDOM from the deep ocean and remineralisation could occur rapidly under the ‘right’ conditions (Baltar et al. 2021; Gonsior et al. 2022; Mo et al. 2025).

The main sources of DOM in the ocean include phytoplankton through exudation and viral lysis (Carlson and Hansell 2014), and allochthonous sources such as terrestrial runoff (Opsahl and Benner 1997) and atmospheric deposition (Jurado et al. 2008). Prokaryotes are considered the main DOM sinks through biomass build‐up and respiration (Azam et al. 1983), but they can also release DOM and transform its composition by various mechanisms (Jiao and Zheng 2011). The transformation of LDOC into RDOC by prokaryotes and its further persistence in the ocean interior has been proposed as a significant C sequestration mechanism termed the MCP (Jiao et al. 2010). The reasons explaining this generation and its persistence in the ocean are, however, still far from being elucidated. RDOM persists in the ocean due to a combination of concentration, chemical structure, bioenergetics and dilute but diverse prokaryotic communities (Moran et al. 2022, Dittmar and Lennart 2024). In the deep ocean, DOM exists at low concentrations (~40 μmol L^−1^) and is structurally diverse with unsaturated and aromatic compounds in different water masses (Flerus et al. 2012; Hansman et al. 2015; Medeiros et al. 2017). Deep DOM composition, along with a diverse prokaryotic community, suggests biochemical pathways capable of oxidising components of the recalcitrant DOM pool and affect DOM processing (Landry et al. 2017; Sebastián et al. 2021). Despite significant advances in understanding the MCP, the DOM processing and persistence of RDOM in the deep ocean remain unclear.

The Mediterranean Sea is the largest semi‐enclosed basin on Earth with strong surface currents and deep water formation that influence the basin's circulation and biogeochemical processes (Christensen et al. 1989; Santinelli et al. 2010). Throughout a given year, phytoplankton‐derived DOM and rivers discharge labile and terrestrial DOM mainly in spring (Para et al. 2010; Gonzalez et al. 2019; Von Jackowski et al. 2024). Subsequently, during summer stratification, prokaryotic communities become limited in nutrients, mostly phosphorus, which may cause an accumulation of labile and SLDOC in surface waters in late summer (Romera‐Castillo et al. 2013; Sánchez‐Pérez et al. 2020; Bouchachi et al. 2025) due to the malfunctioning of the microbial loop, or alternatively, the release of bacterial‐derived DOM during phosphorus limitation (Bouchachi, Obernosterer, Carpaneto Bastos, et al. 2023). Since prokaryotic abundances, production and enzymatic activities remain relatively high in summer (Alonso‐Sáez et al. 2008), we hypothesise that prokaryotes may significantly contribute to an accumulation of DOM, therefore recalcitrant, in the euphotic layer during the summer stratification. The predominance of one of the other processes will determine the lability of accumulated DOM, with consequences on the exported carbon. Wintertime mixing and deep water formation processes transport DOM from the euphotic to the meso‐ and bathypelagic layers, predominantly in the Gulf of Lions and the Adriatic Sea (Schröder et al. 2006; Santinelli et al. 2010, 2015). The vertical export is crucial for carbon sequestration and structuring the prokaryotes community in the deep ocean (Mestre et al. 2018; Sebastián et al. 2021). Thus, seasonal changes in DOM composition will have an impact on its final fate (remineralised vs. accumulated) once it reaches mesopelagic waters after winter mixing.

DOM reactivity fractions can be quantified using biomarkers, such as carbohydrates or amino acids (Davis and Benner 2005). In addition to bulk concentrations, dissolved hydrolysable amino acids (DHAA) can be separated into their enantiomeric forms. L‐DHAA are mostly derived from phytoplankton and D‐DHAA are of prokaryotic origin as key constituents of cell walls (Park and Strominger 1957; Hancock 1960; Lam et al. 2009). Enantiomers of DHAA can be used as biomarkers of DOM lability in the ocean, where L‐DHAA are more labile than their D‐DHAA counterparts and contribute predominantly to the SLDOC and SRDOC pools (Kaiser and Benner 2008), making D‐DHAA good tracers for the MCP. Furthermore, the optical properties of DOM can serve as lability proxies based on its optical properties: chromophoric (or coloured) DOM (CDOM) and fluorescent DOM (FDOM). CDOM is the DOM fraction that absorbs light, while FDOM is the fraction containing compounds in the ultraviolet light spectrum that fluoresce. Fluorescence spectroscopy analyses are used to define different groups of fluorophores that can be assigned to humic‐like or protein‐like groups of compounds and provide information about DOM origin and lability (Coble 1996). In the present study, we focused on FDOM protein‐like compounds (peak‐T) that are indicative of fresh and LDOM (Coble 1996; Nieto‐Cid et al. 2006), while humic‐like FDOM components (peak‐M and peak‐C) are usually indicative of prokaryotic activity and terrestrial origin that accumulate in the deep ocean (Yamashita and Tanoue 2008; Catalá et al. 2015).

A better quantification of the microbial carbon demand and DOM transformations in the ocean is essential to understanding global carbon cycling. Accordingly, we performed a transplant experiment exposing DOM from surface waters in two different periods of the year to mesopelagic prokaryotic communities to better understand the consumption, transformation and production of the DOM pool and whether substrate quality affects prokaryotes' activity and diversity. Our research questions are (a) is the late spring‐DOM more labile than the late summer‐DOM? and (b) does a change in DOM composition affect DOM release by prokaryotes in the Mediterranean Sea?

## 2 Materials and Methods

### 2.1 Experimental Design

**FIGURE 1:**
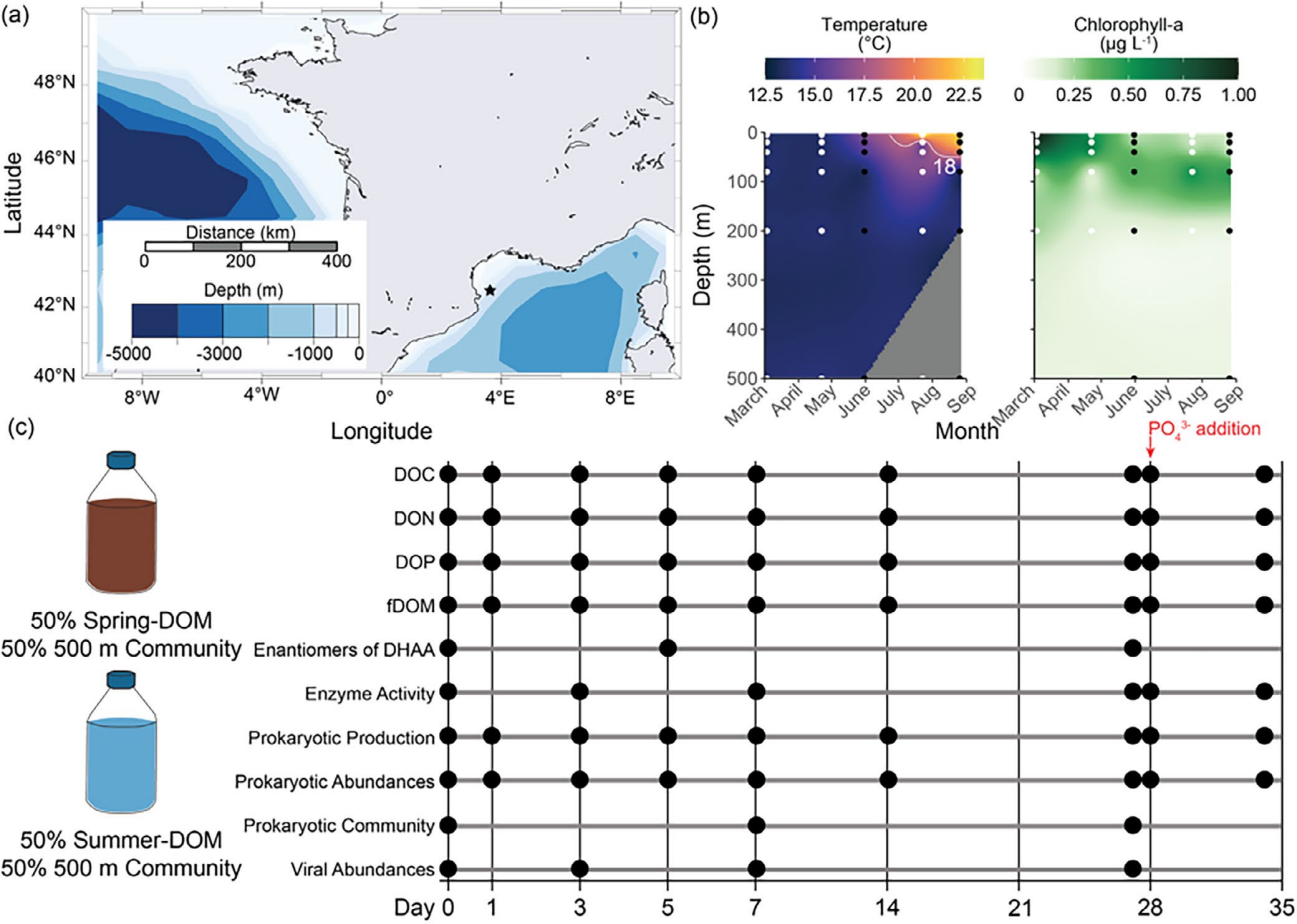
Location and design of the spring‐DOM and summer‐DOM incubation experiments in 2021. The (a) bathymetry with location (star), (b) temperature, and chlorophyll‐a concentrations of Microbial Observatory Laboratory Arago (MOLA). The (c) experiment was set up with surface waters from 31 May (spring‐DOM) and 26 August (summer‐DOM) that were mixed with prokaryotic communities from 500 m and incubated to sample the following parameters: Dissolved organic carbon (DOC), dissolved organic nitrogen (DON), dissolved organic phosphorus (DOP), fluorescent dissolved organic matter (fDOM), dissolved hydrolyzable amino acids (DHAA), among others.

The incubation experiments were centred around the ship‐based time series site ‘Microbial Observatory Laboratory Arago’ (MOLA, 42°27′205 N—03°32′565 E) in the NW Mediterranean Sea (NWMed, Figure S1). Seawater was collected at 5‐m depth to prepare the DOM filtrate on 31 May 2021 (spring‐DOM) and 26 August 2021 (summer‐DOM). To obtain the DOM filtrate, we rinsed the all‐glass filtration system with ~500 mL of ultrapure water to minimise contamination and sequentially filtered through prerinsed 0.8 and 0.2 μm pore‐sized polycarbonate filters using a vacuum pump with low pressure. The DOM filtrate from 31 May 2021 was stored (+4°C) until the start of the experiment. Previous tests showed negligible changes in DOM concentration and minimal prokaryotic growth over a few months (data not shown); anyway, before starting the experiment, spring‐DOM filtrate was re‐filtered through 0.2 μm to exclude any potential interference. On 26 August 2021, DOM filtrate was prepared from 5‐m depth and seawater from 500‐m depth was prefiltered through 0.8 μm pore‐sized polycarbonate filters to remove grazers for prokaryotic inoculum. Incubation experiments were set up as triplicates for each timepoint per treatment of DOM filtrate (‘spring‐DOM’ and ‘summer‐DOM’), mixed with prokaryotic inoculum (50%/50% vol) and incubated in the dark at in situ temperature (+13°C) starting on the 26th of August for 34 days (Figure 1). On Day 27 of 34, once sampling was completed, we resupplied 1.3 μmol PO_4_^3^‐ L^−1^ per bottle since PO_4_^3^ concentrations were at extremely low concentrations the day prior and suspected to be a limiting nutrient.

### 2.2 DOC and Nutrients

Subsamples for DOM and inorganic nutrients were filtered through a double‐layer of combusted GF/F filters (450°C for 8 h, Whatman, USA). Filtrate of ~12–15 mL was collected in combusted glass ampoules, fixed with 10 μL phosphoric acid (pH < 2) and stored in the dark at room temperature prior to being analysed using high‐temperature catalytic oxidation with a TOC‐L (Shimadzu, Japan) to determine the DOC (Benner and Strom 1993). Three to five injections were done for each sample. The mean coefficient of variation was 1.4%. Deep Seawater Reference of 43–45 μmol C L^−1^ provided by the Hansell Laboratory (University of Miami) in sealed glass ampoules were injected every 20 samples to assess the accuracy of the measurements (DSR lot#04‐21 analysed at 45.3 ± 0.8 μmol C L^−1^). Filtrates were submitted to persulfate wet‐oxidation to determine total dissolved nitrogen (TDN) and phosphorus (TDP, Pujo‐Pay and Raimbault, 1994). Analytical replicates were not taken due to volume restrictions, but previous samples on Mediterranean Sea water at the same range of values showed coefficients of variations of 4.2% for TDP and 2.1% for TDN. Additionally, subsamples for inorganic nutrients were collected in polyethylene tubes and stored frozen (−20°C). Technical triplicates were analysed on a continuous flow nutrient analyser (AA3HR Autoanalyser, Bran‐Luebbe, Ireland) to quantify nitrate (NO3−), nitrite (NO2−) and phosphate (PO43−). Subsamples for ammonium (NH4+) were collected into prerinsed tubes and analysed by fluorometry using a QFX Fluorometer (DeNovix, USA). Subsequently, total dissolved and inorganic N and P concentrations were used to derive dissolved organic nitrogen and phosphorus (DON and DOP). DOC, TDN and TDP consumption rates (μm d‐1) were calculated by fitting linear regressions on data over the first 5 days of the incubation.

### 2.3 FDOM

The optical properties of the filtrate were assessed using FDOM. To measure FDOM absorbance, filtered water was inserted into a 10‐mm Quartz cell (Hellma Analytics, Germany) and measured using three repeated measurements on an FP‐8500 Spectrofluorimeter (Jasco, Japan). Blanks were recorded using an ultra‐high purity water reference in every batch of analysis. Fluorescence intensities were reported in Raman units obtained by dividing the fluorescence units by the MilliQ blank peak area (Raman scatter) excited at 350 nm. We characterised fluorophores using the following excitation/emission pairs: 275 nm/310 nm (peak B, protein‐like), 280 nm/350 nm (peak T, protein‐like), 260 nm/450 nm (peak A, humic‐like), 340 nm/440 nm (peak C, terrestrial humic‐like) and 320 nm/410 nm (peak M, marine humic‐like) (Coble 1996). Variation coefficients between analytical replicates go from 0.27% to 1.9%.

### 2.4 DHAA

Filtrate was collected in combusted glass vials (450°C for 8 h) and frozen (−20°C) to determine DHAA. Samples and blanks using ultrapure water were analysed following the protocol by Escoubeyrou and Tremblay (2014). In brief, liquid‐phase hydrochloric acid hydrolysis (30% Suprapure, Merck Millipore, USA; 110°C for 20 h under vacuum) and o‐phthaldialdehyde derivatisation with either N‐Isobutyryl‐L‐cysteine or N‐Isobutyryl‐D‐cysteine (Sigma‐Aldrich, USA) separated enantiomeric amino acids and achiral amino acids by reversed‐phase high‐performance liquid chromatography (Ultimate 3000, Thermo Fisher Scientific, USA) using a Gemini C18 column (Phenomenex, USA). Asparagine and glutamine were deaminated during the HCl‐hydrolysis and quantified as aspartic acid (Asx) and glutamic acid (Glx). Overall, 13 L‐DHAA were detected: alanine, arginine, Asx, Glx, histidine, isoleucine, leucine, lysine, phenylalanine, serine, threonine, tyrosine and valine. A total of 6 d‐DHAA were detected: alanine, Asx, Glx, leucine, serine and valine. Additionally, beta (b‐)alanine, glycine and gamma‐aminobutyric acid (GABA) were detected. Analytical replicates were not taken, but replicate measurements of standards show variation coefficients of 5.6% for all DHAA and 1.3% for D‐DHAA. The average variation coefficient for each individual amino acid can be found in Escoubeyrou and Tremblay (2014). Individual DHAAs were used to calculate the degradation index (Dauwe et al. 1999) and reactivity index (Equation 1; Gaye et al. 2022).

(1) $$\text{Reactivity index}=\left(\mathrm{tyr}+\mathrm{phe}\right)/\left(\text{gaba}+\text{bala}\right)$$

### 2.5 Prokaryotic and Viral Abundances

Subsamples were collected for flow cytometry to determine prokaryotic and viral abundances (del Giorgio et al. 1996). Unfiltered water was collected in 2 mL tubes, fixed with 85 μL glutaraldehyde at 1% final concentration, incubated for 15 min at room temperature, flash frozen and stored frozen (−80°C). For prokaryotic cell counts, the thawed samples were incubated in the dark with SybrGreenI (S7585, Invitrogen, Thermo Fisher Scientific) for 15 min and, once injected, counted by detecting the DNA‐binding dye on a Cytoflex (Beckman Coulter, USA) on side scatter versus green fluorescence plots in low flux. The instrument was calibrated with TruCount beads (Becton Dickinson, USA). Cell abundances were estimated after visual inspection and manual gating in the cytogram using the software FlowJo (v7.6) (Becton Dickinson, USA). Counts of virus and prokaryotes from fixed samples were performed by flow cytometry using the SYBR Green I dye (S7585, Invitrogen, Thermo Fisher Scientific) according to (Brussaard 2004) with a BD FACSAria Fusion SORP (BD Sciences, San Jose, CA, USA) equipped with an air‐cooled laser delivering 50 mW at 488 nm with 502 longpass, and 530/30 bandpass filter set‐up. Virus counts were acquired and analysed with BD FACSDiva 9.0 software.

### 2.6 Prokaryotic Production and Extracellular Enzyme Activity

Subsamples were collected to determine prokaryotic heterotrophic production (PHP) using the microcentrifuge method (Smith and Azam 1992). Triplicate samples and one killed control (1.5 mL each) were labelled using 3H‐leucine (Perkin Elmer, USA) at a final concentration of 20 nmol L^−1^. The samples were incubated for 2 h in the dark at +13°C and terminated using trichloroacetic acid (TCA) at a final concentration of 5%. Leucine incorporation was converted into PHP by applying a factor of 0.54 kg C mol leucine^−1^ and assuming no intracellular isotope dilution (Giering and Evans 2022).

Subsamples were collected to determine extracellular enzyme activities. Unfiltered water was pipetted in triplicate into 96‐well black plates and 4‐methylumbelliferyl‐butyrate was added for esterase, 4‐methylumbelliferyl phosphate for alkaline phosphatase (APase), and L‐leucine‐7‐amido‐4‐methyl coumarin for leu‐aminopeptidase (AMA) at a final concentration of 125 μmol L^−1^. The fluorescence of fluorogenic substrates was measured on a plate reader of an FP‐8500 Spectrofluorimeter (Jasco, Japan) at 365 nm/450 nm (ex/em) wavelengths. Immediately after the addition of the substrates, after 1.5 h, and after 3 to 4 h of incubation in the dark at in situ temperature (+13°C). The increase of fluorescence units during the period of incubation was converted into enzymatic activity (μM h‐1) with standard curves prepared with 7‐amido‐4‐methylcoumarin for AMA and 4‐methylumbelliferone for APase and esterase (Hoppe 1983).

### 2.7 Prokaryotic Community Composition

Subsamples were collected to characterise the prokaryotic community composition. 200 mL of water was filtered through 0.22‐μm filters (Nucleopore, USA) using a peristaltic pump and stored frozen (−80°C). The genomic DNA was lysed with lysozyme (20 mg mL‐1, 45 min at 37°C, Sigma‐Aldrich, USA), proteinase K (20 mg mL‐1, 60 min at 55°C, Sigma‐Aldrich, USA), and extracted using the Zymobionics DNATM Miniprep Kit (Cat. No.: D6005, Zymo Research, USA). PCR‐amplification using the universal 16S rRNA 515F and 926R primer pair covering the v4‐v5 hypervariable region (Parada et al. 2016) and sequenced 2 × 300 bp on a MiSeq platform (Illumina, USA) at LGC Genomics GmbH (Berlin, Germany).

Amplicon reads were processed in R‐Studio. FastQ files were processed into amplicon sequence variants (ASVs) using the ‘dada2’ (v1.16) package (Callahan et al. 2016). Filtering settings were truncLen = c(220,220), maxN = 0, minQ = 2, maxEE = c(2,2) and truncQ = 0, followed by merging using minOverlap = 10 and chimera removal. After singleton removal, we obtained an average of 35 k reads per sample. ASVs were taxonomically classified using the Genome Taxonomy Database (GTDB) (Parks et al. 2018). ASVs with less than three counts in less than 3% of samples were excluded.

### 2.8 Statistical Analyses

Data analyses and visualisations were conducted in RStudio (v2023.09.1+, [R Core team 2024]). For all environmental parameters, the average was calculated using the arithmetic mean of the timepoint per treatment and outliers were determined using a very restrictive z‐score of greater than +1.0 or less than −1.0, which was equivalent to approximately 68% standard deviation. Spearman correlations, analysis of variance (ANOVA), and Tukey Honest Significant Difference tests were conducted using the stats (v4.2.1) and dplyr (v1.1.4 [Wickham H, François R, Henry L, Müller K, Vaughan D {2023}]. dplyr: A Grammar of Data Manipulation. R package version 1.1.4, https://github.com/tidyverse/dplyr, https://dplyr.tidyverse.org).

## 3 Results

### 3.1 Prokaryotic and Viral Abundances

**FIGURE 2:**
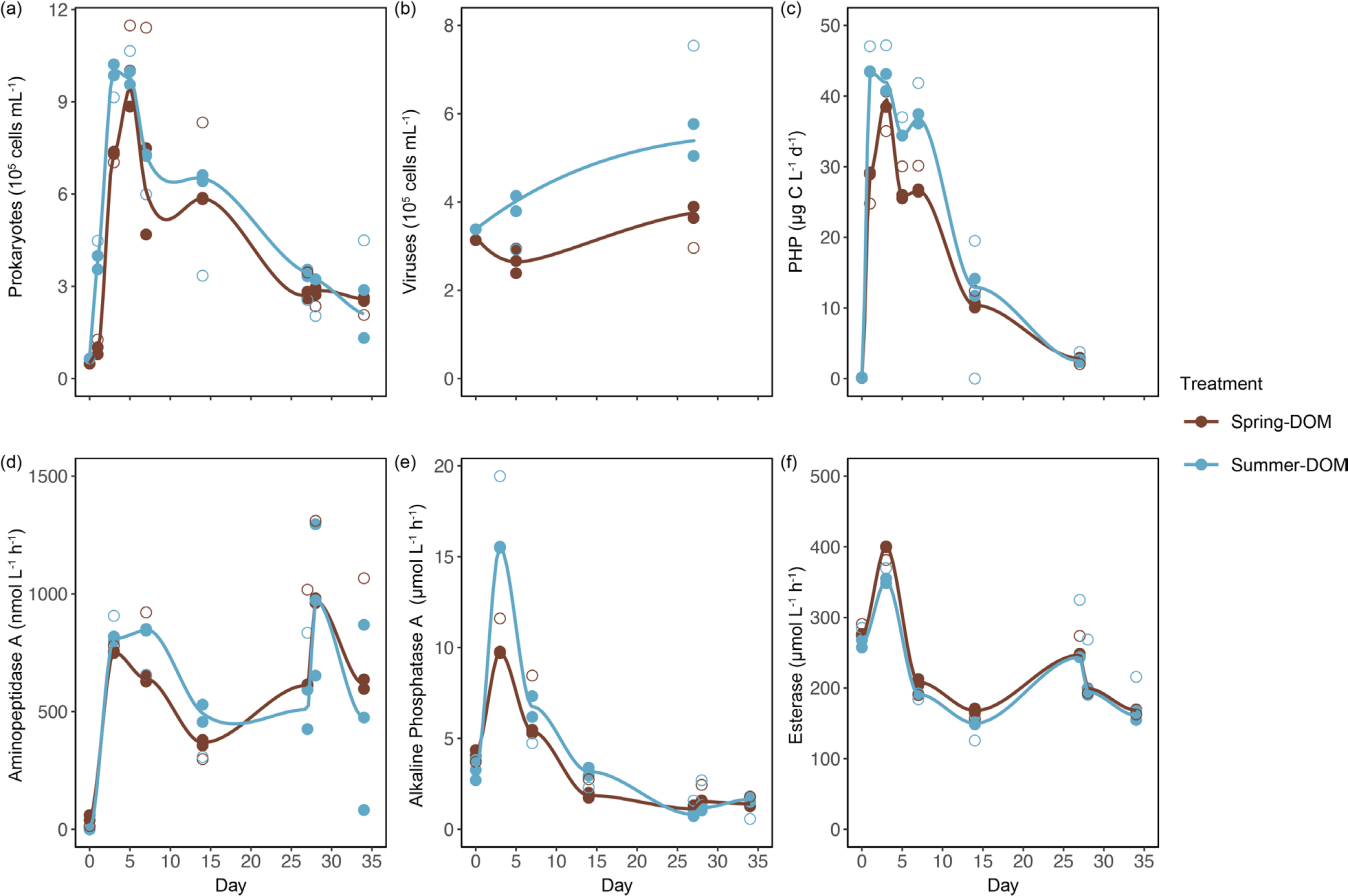
Enrichment experimental changes in prokaryotic abundance and production, viral abundances and enzymes. The (a) prokaryotic abundance, (b) prokaryotic heterotrophic production, (c) viral abundance, (d) aminopeptidase, (e) alkaline phosphatase and (f) esterase.

The prokaryotic abundances peaked early in both incubations between Days 0 and 5, while the viral abundances tended to increase in both incubation experiments. On Day 3, prokaryotic cells reached maximum abundances in summer‐DOM treatment (10 ± 0.25 × 10^5^ cells mL^−1^, *n* = 3; Figure 2), which were followed by maximum abundances of 9.4 ± 0.83 × 10^5^ cells mL^−1^ on Day 5 in spring‐DOM (*n* = 3; Figure 2). After the peaks in abundance, prokaryotic abundances decreased around 3.7‐fold in spring‐DOM and 4.8‐fold in the summer‐DOM treatment until the end of the incubation experiment without an apparent response to the replenished phosphorus concentrations on Day 28. Conversely, viral abundances decreased by 18% in spring‐DOM, while increasing by 7% in the summer‐DOM treatment between Days 0 and 5. Between Days 5 and 27, viral abundances increased by 32% in the spring‐DOM and a significant increase by 69% in the summer‐DOM treatment (*r* = 0.95, *p* < 0.05).

### 3.2 Prokaryotic Production and Enzymatic Activity

PHP peaked between Days 0 and 3 in both treatments, with consistently higher PHP in summer‐DOM treatment. In the spring‐DOM treatment, PHP was 40 ± 1.6 μg C L^−1^ d^−1^ (*n* = 3), while it was 43 ± 0.052 nmol L^−1^ h^−1^ (*n* = 3) in the summer‐DOM treatment. PHP remained higher in the summer‐DOM treatment on Day 5 and on, despite a decrease in prokaryote abundances. Enzymatic activities showed more variability throughout the incubation experiments (Figure 2). Between Days 0 and 3, we observed a growth phase in both incubations. On Day 3, esterase was significantly higher in spring‐DOM (400 ± 0.82 μmol L^−1^ h^−1^ [*n* = 3]) compared to summer‐DOM treatment (352 ± 4.8 μmol L^−1^ h^−1^ [*n* = 3], one‐way ANOVA, *p* = 0.016), whereas APase was significantly higher in summer‐DOM (16 ± 0.045 μmol L^−1^ h^−1^ [*n* = 3]) compared to spring‐DOM treatment (9.7 ± 0.058 μmol L^−1^ h^−1^ [*n* = 3], one‐way ANOVA, *p* = 0.011). AAMA was also higher in summer‐DOM (809 ± 14 μmol L^−1^ h^−1^ [*n* = 3]) than in spring‐DOM (753 ± 5.7 nmol L^−1^ h^−1^ [*n* = 3]). Esterase and APase significantly decreased throughout both incubation experiments (*r* < −0.59, *p* < 0.05). The replenished phosphorus concentrations on Day 28 did not affect esterase activity but did temporarily increase AMA and APase in both amended treatments (Figure 2).

### 3.3 Prokaryotic Diversity and Community Composition

**FIGURE 3:**
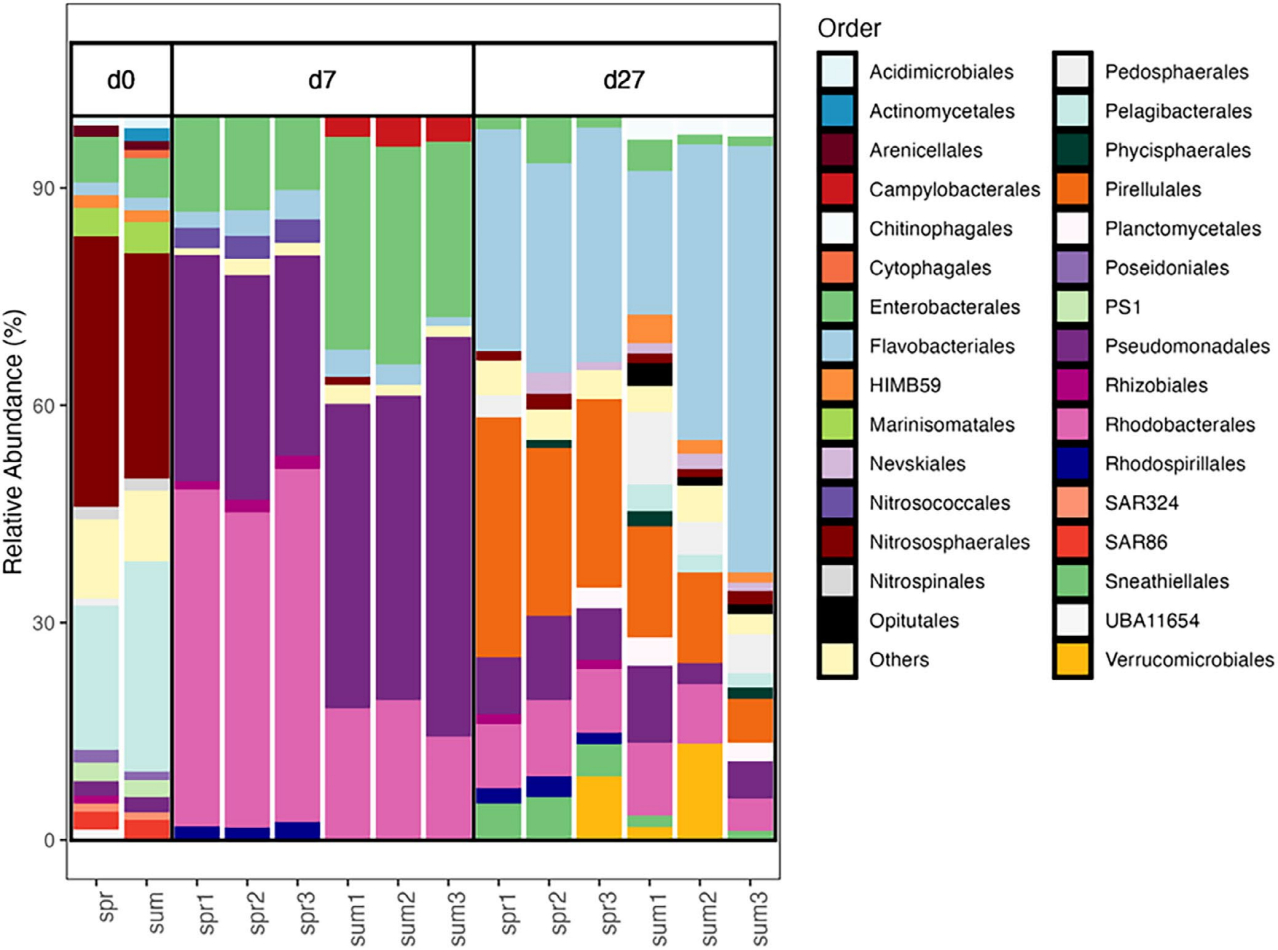
Experimental changes in relative abundance of the main prokaryotic orders.

At t0, the community composition was dominated by Nitrososphaerales and Pelagibacterales (Figure 3). Between Days 0 and 7, we observed a significant decrease in the observed richness (Tukey Test, *p*_adj_ < 0.01), Shannon‐Wiener index (Tukey Test, *p*_adj_ < 0.05) and Simpson‐Diversity Index (Tukey Test, *p*_adj_ < 0.001) in the spring‐DOM and summer‐DOM treatment (Figure S2). Enterobacterales, Pseudomonadales and Rhodobacterales dominated both treatments (Figure 3), but Rhodospirillales were enriched with relative abundances of 2% in the spring‐DOM treatment, while Campylobacterales were enriched with relative abundances of 4% in the summer‐DOM treatment (Figure 3). At the ASV level, unclassified Rhodobacteraceae (order Rhodobacterales) was the most abundant (36%) and significantly enriched ASV in the spring‐DOM treatment (ANOVA; *p*_adj_ < 0.001), followed by *Marinobacter* (8%), from the order Pseudomonadales, while the summer‐DOM treatment was significantly enriched in members of the genus *Marinomonas* (order Pseudomonadales, 28%), *Vibrio* (8%) and *Pseudoalteromonas* (5%) (order Enterobacterales) (ANOVA, *p* < 0.05; Figure S3). The community composition changed between Days 7 and 27 following the decrease in cell abundance, since treatments shifted to a dominance of Flavobacteriales and Pirellulales (Figure 3). Pirellulales were particularly present with 25% relative abundances in the spring‐DOM treatment, while the summer‐DOM treatment was enriched in Pedosphaerales (6%), Chitinophagales (3%) and HIMB59 (2%) (Figure 3). At the ASV level, the spring‐DOM treatment was significantly enriched in *Rhodopirellulla‐B* (20%, order Pirellulales) and an unclassified member of Flavobacteriales (13%) (Figure S3), while summer‐DOM was enriched in *Tenacibaculum* (5%, order Flavobacteriales) and AAA164‐E04 (4%, order Pedosphaerales) (Figure S3).

### 3.4 Changes in Dissolved Organic Carbon and Nutrients

**FIGURE 4:**
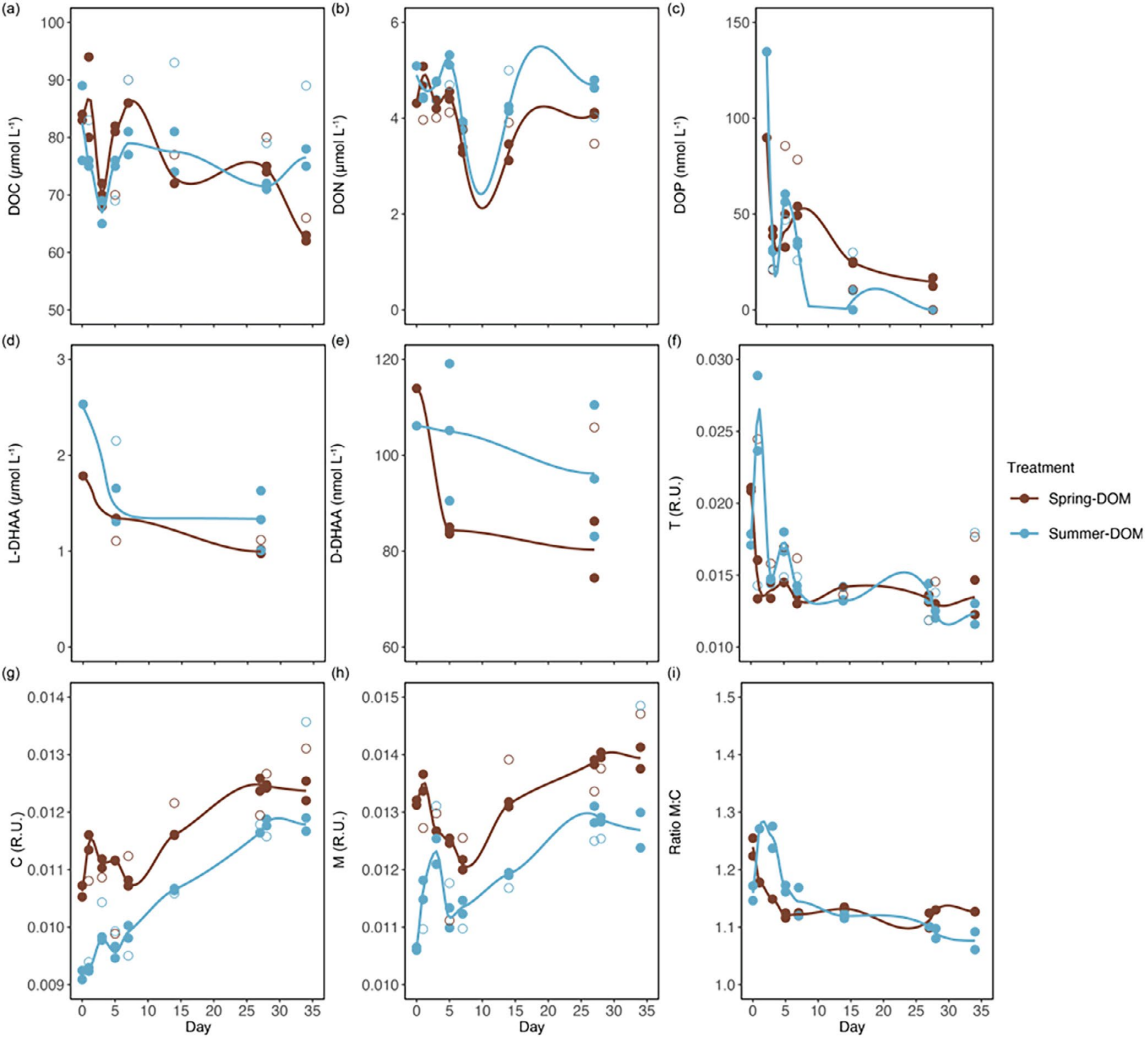
Experimental changes in dissolved organic matter. The (a) dissolved organic carbon (DOC), (b) dissolved organic nitrogen (DON) and (c) dissolved organic phosphorus (DOP). From dissolved hydrolysable amino acids (DHAA), the (d) L‐enantiomers and (e) D‐enantiomers. Additionally, specific FDOM emission and excitation wavelengths correspond to the (f) protein‐like (peak T), (g) humic‐like (peak C), (h) humic‐like (peak M) fluorescence components and (i) ratio of M to C.

Nutrients and DOM fluctuated throughout the incubation experiments with differences between treatments. At t0, inorganic nutrients and DOC showed < 2% difference between the treatments, while DON was 18% higher and DOP was 50% higher in the summer‐DOM than spring‐DOM treatment (*n* = 2; Table S1). Accordingly, DOC:DON:DOP were 934:48:1 in spring‐DOM and 564:38:1 in summer‐DOM at t0. During the active growth phase, TDN was consumed at a higher rate in the spring‐DOM treatment (0.17 ± 0.05 μmol L^−1^ d^−1^) than in the summer‐DOM treatment (0.09 ± 0.07 μmol L^−1^ d^−1^). In the summer‐DOM treatment, the decrease in TDN was followed by an increase until the end of the incubation experiment (Figure 4). In contrast, TDP declined significantly in both treatments (Tukey Test, *p* < 0.05) but at a higher decrease in the summer‐DOM (0.023 ± 0.001 μmol L^−1^ d^−1^) than in the spring‐DOM (0.013 μmol L^−1^ ± 0.003 μmol L^−1^ d^−1^, Table 1). Consequently, during the growth phase, prokaryotes took up nutrients at lower C:P and N:P ratios in the summer‐DOM treatment (Table 1).

**TABLE 1:** Consumption of dissolved organic matter during the active growth phase (d0‐d3) of the incubation: Arithmetic means and standard deviation of dissolved organic carbon (DOC), total dissolved nitrogen (TDN), total dissolved phosphorus (TDP) and ratios.

Consumption	Spring‐DOM		Summer‐DOM	
	Mean	SD	Mean	SD
DOC (umol L‐1 d‐1)	1.84	1.86	1.92	0.640
TDN (umol L‐1 d‐1)	0.17	0.05	0.09	0.067
TDP (umol L‐1 d‐1)	0.013	0.003	0.023	0.001
CN	14.52	18.36	34.57	31.696
CP	161.35	180.30	84.70	29.257
NP	13.46	4.24	3.98	3.058

Changes in total nutrients were somewhat followed by inorganic nutrient dynamics. Dissolved inorganic nitrogen (DIN) decreased significantly throughout spring‐DOM (*r* = −0.89, *p* < 0.001) and summer DOM treatments (*r* = −0.56, *p* < 0.05). NH_4_+ decreased significantly in both treatments between Days 1 and 3 (one‐way ANOVA, *p* < 0.01) prior to fluctuating in the spring‐DOM and increasing in the summer‐DOM on Day 14 (Figure S4). DIP decreased between Days 0 and 5 in spring‐DOM but between Days 0 and 3 in summer‐DOM (Figure S4). Furthermore, DIP significantly correlated with prokaryotic abundances in both treatments (*r* < −0.83, *p* < 0.05) but only with PHP and APase in the summer‐DOM treatment (*r* < −0.79, *p* < 0.01). DOP significantly decreased in both treatments (*r* < −0.75, *p* < 0.01), particularly in summer‐DOM (Tukey Test, *p* < 0.01), where concentrations also reached below the detection limits from Day 14 (Figure 4c) and suggests a higher demand for DOP in the summer‐DOM treatment.

### 3.5 Changes in DOM Composition

Temporal changes were observed in DOM bioavailability with a decrease in the reactivity index (based on DHAA) in both treatments, while the degradation index remained stable (spring‐DOM) or increased then decreased (summer‐DOM); but showed poor replicability in both treatments (Figure S5). At t0, DHAA represented 3% and 4% of DOC in the spring‐DOM and summer‐DOM treatments respectively. D‐DHAA concentrations were 7% higher in the spring‐DOM, whereas L‐DHAA was 42% higher in the summer‐DOM treatment at t0 (*n* = 1; Figure 3, Table 1). During the incubation, L‐enantiomers of DHAA decreased markedly in both treatments (Figure 4d) along with D‐DHAA that decreased in the spring‐DOM, while D‐DHAA was stable in summer (Figure 4e). Looking at individual amino acids, in the spring treatment, L‐Ser, L‐Gly and L‐Lys were the most consumed (> 55% during the first 5 days), while L‐Glu, L‐His and L‐Ser (> 50% during the first 5 days, Figure S6).

The FDOM excitation‐emission peak corresponding to peak‐T (protein‐like) was 20% higher and peak‐C (humic‐like) was 16% higher in spring‐DOM than summer‐DOM treatment at t0, which was likely linked to more photodegradation in summer (Table S1). The protein‐like (peak‐T) fluorescence decreased in both treatments (Figure 4f) at 1.05 × 10^−4^ ± 5 × 10^−5^ R.U. d^−1^, while humic‐like (peak‐C) fluorescence gradually accumulated in both treatments over time (Figure 4g) but at a higher rate in the summer‐DOM treatment (8.83 × 10^−5^ ± 6.1 × 10^−6^ R.U. d^−1^) than in the spring‐DOM (5.68 × 10^−5^ ± 6.4 × 10^−6^ R.U. d^−1^). Conversely, another humic‐like fluorescence component (peak‐M), 19% higher in spring‐DOM treatment at t0, only increased in the summer‐DOM treatment coinciding with the bacterial growth phase (Figure 4h), thus an increase in the Peak‐M/Peak‐C ratio could be observed in this phase in summer‐DOM with a subsequent decrease in both treatments after Day 5 (Figure 4i).

## 4 Discussion

Our experiment simulated the possible factors controlling DOM consumption and release by the deep ocean prokaryotic communities in the Mediterranean Sea. Initially, we hypothesised that spring vs. summer DOM would differ in their availability to deep microbes and that prokaryotes would significantly contribute to an accumulation of recalcitrant DOM, which we confirmed with our incubation experiments. The differences in initial substrate composition affected DOM release, similar to previous studies (Jorgensen et al. 2014; Koch et al. 2014; Aparicio et al. 2015). Furthermore, we demonstrated that higher DOM concentrations coincide with high total phosphorus (TDP) demand, elevated APase activity and higher viral abundances (Figure 1, Table 1).

### 4.1 Seasonal Changes in DOM Bioavailability in MOLA

Abiotic factors control the niche preferences in the prokaryotic community and affect the seasonal changes in the DOM pool. At t0 of our incubation experiment, differences in the DOC:DON:DOP ratios, 50% higher DOP and 42% higher L‐DHAA concentrations showed that DOM was more labile in the summer‐DOM than the spring‐DOM treatment, which is uncommon for the seasonal DOM composition at the MOLA time series. Typically, the decay of the phytoplankton spring bloom enriches surface waters in LDOM and SLDOM compounds, such as carbohydrates, phytoplankton‐derived carbohydrate‐like transparent exopolymeric particles (TEP), and amino acids between February and May each year (Jones et al. 2013; Ortega‐Retuerta et al. 2018; Gonzalez et al. 2019; Von Jackowski et al. 2024). Then, photodegradation of DOM and prokaryotic processing of labile compounds builds the pool of recalcitrant compounds in summer (Vila‐Reixach et al. 2012; Romera‐Castillo et al. 2013; Bouchachi et al. 2025). Additionally, river discharge of low salinity waters (LSWs) carries LDOM promoting PHP that also contribute to the pool of recalcitrant compounds in summer (Laghdass et al. 2010; Gonzalez et al. 2019). However, the unexpected mismatch in lability compared to previous observations from the MOLA time series, which precluded us from validating our initial hypothesis, was likely due to an episode of elevated haptophytes biomass in August 2021 (Von Jackowski et al. 2024). Phytoplankton biomass is known to vary interannually at MOLA (Von Jackowski et al. 2024), as well as other environmental disturbances in the NWMed, such as storms (Barrillon et al. 2023), dust deposition (Ternon et al. 2010), heatwaves (Juza et al. 2022) and other episodic events the effects of which need to be elucidated in coming years. We cannot exclude the fact that water manipulations to set up the experiments can slightly change initial DOM concentration and composition, for example, filtering, even if we kept a low vacuum pressure, may break some cells leading to the release of some labile compounds. We strongly encourage future studies to focus on both DOM dynamics and microbial communities during successive years, in combination with repeated experiments, to study whether seasonal DOM accumulation involves a change from labile to recalcitrant DOM.

### 4.2 Consumption, Release and Accumulation of DOM

Initial differences in DOM composition inoculated with the same prokaryotic community triggered different requirements in C, N and P demands that affected DOM accumulation patterns (Table 1, Figure 4). We observed that phosphate spikes on Day 27 did not substantially affect these DOM accumulation patterns or cell abundances (Figure 2), thus demonstrating that DOM at that time was composed mostly of refractory or semi‐labile compounds and its accumulation was not due to the inability of prokaryotes to use it when P limited. DOC, DON and DOP fluctuated throughout both treatments, particularly from Day 0 to 14, which might reflect a consumption but also a release of C‐ and N‐containing DOM compounds (Ogawa et al. 2001; Kaiser and Benner 2008; Ortega‐Retuerta et al. 2021). We observed almost a continuous increase in humic‐like FDOM in both treatments but at a higher rate in the summer‐DOM treatment, along with a relative increase in the proportion of D‐DHAA (Figure 4). In addition, prokaryotes, mainly in the summer‐DOM treatment, actively grew and consumed N‐containing compounds during active growth, but released N‐containing compounds (TDN, NH4+ and D‐DHAA) during cell decrease. During the cell decay incubation phase, PHP was systematically higher in the summer‐DOM than in the spring‐DOM, which was not accompanied by higher prokaryotic abundances (Figure 2). Therefore, per‐cell prokaryotic production was higher at the summer‐DOM treatment than in spring‐DOM during the cell decay phase, suggesting that prokaryotes did not effectively incorporate carbon into biomass and instead released carbon into the medium. Higher P demand during active growth in the summer‐DOM treatment (Table 1) could have led to an excess in N in the cells that would have been released to the medium, explaining the higher accumulation of NH4+, TDN and D‐DHAA in this condition.

Prokaryotes consumed P‐containing DOM compounds that were linked to APase activities and resulted in an accumulation of semi‐labile to RDOM during the active growth phase. Particularly in the summer‐DOM treatment, APase correlated with DIP (*r* = −0.97, *p* = 0.0012) and suggested that initial DOP concentrations enhanced APase activity. An alternative nonexclusive explanation for higher APase in the summer‐DOM treatment could be the presence of dissolved APase in the summer‐DOM extract since this enzyme has a long lifetime in seawater over 16 days (Thomson et al. 2019) and APase activities in the NWMed are higher in late summer (Zaccone et al. 2002). The observed accumulation of humic‐like FDOM is consistent with previous studies and confirms that humic‐like FDOM compounds are good tracers of RDOM (e.g., [Catalá et al. 2015; Lønborg et al. 2015]). The observed succession of different FDOM humic‐like components in our incubations, this is the release of peak M that was progressively replaced by peak C (Figure 4), suggests that peak M is a byproduct of active cell growth under some conditions and can be consumed (Bouchachi, Obernosterer, Carpaneto Bastos, et al. 2023), while peak C is a relatively more RDOM pool and persists after microbial processing. Peaks M and C have been identified as RDOM persisting in the ocean for years (Catalá et al. 2015) and covary in previous studies looking at FDOM changes in microbial incubations (Shimotori et al. 2009; Arai et al. 2017; Grunert et al. 2021; Gómez‐Letona et al. 2022). However, component‐M peaked earlier than component‐C during deep‐sea incubations (LaBrie et al. 2022). In addition, in line with our observations, Shimotori et al. (2009) reported shifts in bacterial‐derived FDOM, from an enrichment in compounds excited at 310 nm excitation to compounds excited at > 330 nm, following bacterial growth phases. More studies are needed to identify the processes associated with the production of the different DOM humic‐like components in the ocean RDOM pool.

Moreover, few published studies combine changes in enzyme activities and FDOM. For example, APase and prokaryotic growth were associated with higher protein‐like DOM persistence (Steen et al. 2016), determined the release of carbon‐rich DOM that forms mucilage (Danovaro et al. 2005), and were linked to P‐demand that resulted in DOC accumulation (Malfatti et al. 2014). P‐limitation enhances the release of humic‐like FDOM (Bouchachi, Obernosterer, Marie, et al. 2023), which is somewhat contrary to our findings since higher humic‐like FDOM is produced when more P is available at t0. Regardless, initial N:P ratios of 33:1 in spring‐DOM and 36:1 in summer‐DOM suggest that phosphorus was the limiting nutrient in our incubations and that a surplus in C and N and the use of organic P via the production of APase are the best predictors of RDOM release. Overall, the succession in bacterial‐derived FDOM compounds remains unclear and we encourage future studies to identify the processes associated with the production of the different DOM humic‐like components in the ocean RDOC pool.

### 4.3 Impact of Viruses on DOM Accumulation

Viruses and virus‐like particles (VLPs) became more abundant and might have contributed to an accumulation of humic‐like DOM. It is possible that the inclusion of viruses was a systematic error, since viruses were included in the initial filtrate for DOM additions, and viruses are generally more abundant in the Mediterranean Sea in late summer than in spring (Magiopoulos and Pitta 2012). Alternatively, differences in DOM composition and prokaryotic community could affect the vulnerability of prokaryotes to viral infections (Töpper et al. 2011; Liu et al. 2017). Viral lysates can be incorporated into bacterial biomass, which later result in the release of combined amino acids, predominantly the D‐enantiomers of DHAA, during viral lysis (Noble and Fuhrman 1999; Middelboe and Jørgensen 2006). Thus, higher viral lysis in the summer‐DOM treatment could also explain the higher increase in the proportion of d‐DHAA in summer (Figure 4). Furthermore, viral lysis can result in an accumulation of humic‐like FDOM (Xiao et al. 2021) and enhance the bacterial transformation of viral lysates of picocyanobacteria into refractory compounds, thereby stimulating the MCP (Zhao et al. 2019).

### 4.4 Prokaryotic Community Members Responsible for DOM Transformation and Accumulation

Seasonal changes in DOM composition promote the development of specific prokaryotic communities and metabolisms adapted to specific DOM compounds. The prokaryotic community shifted from Thaumarchaeota to Flavobacteriales and Pirellulales between Day 0 and 7 (Figure 3). The compositional shift is similar to bottle incubations with mesopelagic water of the Mediterranean Sea (La Cono et al. 2015; Sebastián et al. 2021), but differs from incubations of the Labrador Sea where archaeal members continued to dominate possibly due to the lower temperatures (LaBrie et al. 2022). The dominance of fast‐growing Enterobacterales (genus *Alteromonas* and *Vibrio*), Rhodobacterales and Pseudomonadales (genus *Marinomonas*) at the end of the active growth phase is likely adapted to the utilisation of transient labile organic matter (Duret et al. 2019). *Vibrio* (Enterobacterales) and *Marinomonas* (Pseudomonadales) were the two most abundant ASV's relatively enriched in the summer‐DOM treatment (Figure S3). Both host APase genes (phoX), and specifically *Vibrio* has been linked to APase activity, possibly linked to P degradation in our experiment, while *Marinomonas* may have been responsible for the higher humic‐like accumulation in the summer‐DOM treatment (Kathuria and Martiny 2011; Shimotori et al. 2012; Ortega‐Retuerta et al. 2021). The higher proportion of Pseudomonadales in the summer‐DOM treatment is similar to the proportion of Oceanospirillales (same group but different names in SILVA vs. GTDB databases) in deep DOM incubation experiments (Sebastián et al. 2021), which could suggest a higher resemblance of our summer DOM to deep DOM. In contrast to our results, these authors observed lower FDOM accumulation in those treatments where Pseudomonadales are proportionally more abundant, but Pseudomonadales dominate N‐amended incubations where FDOM was produced, which suggests that these bacterial members produce FDOM (Goldberg et al. 2017). Community composition showed an enrichment of HIMB59 (Pelagibacterales) in the summer‐DOM treatment between Day 7 and 27 (Figure 3). HIMB59 is metabolically distinct from other Alphaproteobacteria in its potential to transport and utilise a broader range of sugars as well as in the transport of trace metals and thiamin (Getz et al. 2023). The versatility in transporters could be an advantage in processing LDOM and simultaneously coexist with more processed prokaryotic‐derived DOM (Figure 3). Alongside HIMB59, *AAA164‐E04* (order Pedosphaerales, class Verrucomicrobia) and *Tenacibaculum* (Flavobacteriales) were present in the summer‐DOM treatment on Day 27 (Figure 3), which are typically present in the particle‐attached fraction or form aggregates (Crespo et al. 2013; Bachmann et al. 2018; Heins and Harder 2022). Although macroaggregates were not present in the incubation experiment (personal observation), microaggregates could have been formed in the summer‐DOM treatment as DOM aggregation has been associated with phosphorus depletion (Lancelot 1995). Alternatively, initial substrate quality could have also affected the aggregation potential (i.e., stickiness) of bacterial‐derived DOM, which has important implications in carbon fluxes.

## 5 Conclusion

Our incubation experiment demonstrates the importance of studying the effect of seasonal DOM dynamics on its prokaryotic processing. We set up the incubation experiment in the context of the MOLA time series with atypical DOM filtrates of a more refractory spring‐DOM treatment and more labile summer‐DOM treatment that was likely due to an unusual haptophyte bloom in August 2021. These results highlight the need for further experiments in successive years to robustly establish the link between temporal changes in DOM composition and its potential availability to prokaryotes. However, our results demonstrate that changes in initial DOM composition determine prokaryotic growth and release of DOM compounds prone to enter the RDOC pool. Based on our observations, we propose a strong link between FDOM accumulation and prokaryotic P‐demand, APase activity and viral lysis in the deep ocean that needs to be studied in the future. In particular, the impact of APase activity on FDOM accumulation has been shown to dominate extracellular enzyme activities in the meso‐ and bathypelagic oceans (Hoppe and Ullrich 1999; Baltar et al. 2009) as well as viral lysis that dominates prokaryotic mortality in the deep ocean (Lara et al. 2017) releasing prokaryotic‐derived DOM possibly relevant in carbon sequestration mechanisms. Prokaryotic processing and FDOM accumulation are subject to spatial and temporal changes as transient increases in labile organic matter are linked to convective processes or the arrival of sinking particles, which can enhance the growth of fast‐growing taxa with high P requirements and the ability to use organic P, thus stimulating the MCP. In the future, the increasing impact of anthropogenic climate change will make assessments of prokaryotic DOM processing in the MCP challenging. Climate change‐associated events, such as marine heat waves, have devastating impacts on marine ecosystems and the interannual variability from the euphotic to bathypelagic zones in the ocean. Therefore, further studies are needed to understand how interannual changes affect microbial functionalities connected with changes in DOM composition.

## Author Contributions

**Eva Ortega‐Retuerta:** conceptualisation, methodology, investigation, validation, resources, writing – original draft, review and editing, supervision, project administration, funding acquisition. **Nawal Bouchachi:** methodology, investigation. **Olivier Crispi:** investigation. **Barbara Marie:** investigation. **Rebeca Campos:** investigation. **Charles‐Hubert Paulin:** investigation. **Karine Escoubeyrou: i**nvestigation, supervision. **Jonathan Colombet:** investigation. **Telesphore Sime‐Ngando:** investigation, funding acquisition. **Anabel Von Jackowski:** conceptualisation, formal analysis, data curation, visualisation, writing – original draft, review and editing.

## Funding

This work was supported by the Agence Nationale de la Recherche (ANR‐20‐CE01‐0007, ANR‐10‐INBS‐02), the Institut National des Sciences de l'Univers (ODISEA, LEFE/CYBER 2019), the Ecole Doctorale 129 and the Moose‐ILICO.

## Conflicts of Interest

The authors declare no conflicts of interest.

## Supporting information


**Data S1:** emi470288‐sup‐0001‐Supinfo.docx.

## Data Availability

Environmental data are uploaded to PANGAEA under the accession number (submitted to be determined). Raw fastq files of 16 s sequences have been deposited at ENA under the accession number PRJEB77937.
