# Supplementary material for: Changes in DOM Quality Determine Prokaryotic Activities and Extracellular Release in the NW Mediterranean Sea: An Experimental Approach
Source: Environ Microbiol Rep. 2026 May 7;18(3):e70288. doi: 10.1111/1758-2229.70288 (PMC13150484; doi:10.1111/1758-2229.70288)
Supplement: Supplementary file 1 — Data S1: emi470288‐sup‐0001‐Supinfo.docx. [file EMI4-18-e70288-s001.docx]

**Supplementary material. Temporal changes in DOM quality determine prokaryotic activities and extracellular release in the NW Mediterranean Sea: An Experimental Approach**

Eva Ortega-Retuerta^1^, Nawal Bouchachi^1^, Rebeca Campos^1^, Olivier Crispi^1^, Barbara Marie^1^, Charles-Hubert Paulin^2^, Karine Escoubeyrou^2^, Jonathan Colombet^3^, Telesphore Sime-Ngando^3^, Anabel von Jackowski^1^

^1^ CNRS/Sorbonne Université, Laboratoire d’Océanographie Microbienne UMR7621, Banyuls sur Mer, France

^2^ Oceanographic Observatory of Banyuls sur Mer (OOB), Banyuls sur Mer, France

^3^ CNRS Laboratoire Microorganismes: Génome Environnement UMR 6023, Aubi​​ère, France

# **Supplementary Figures and Tables**

**
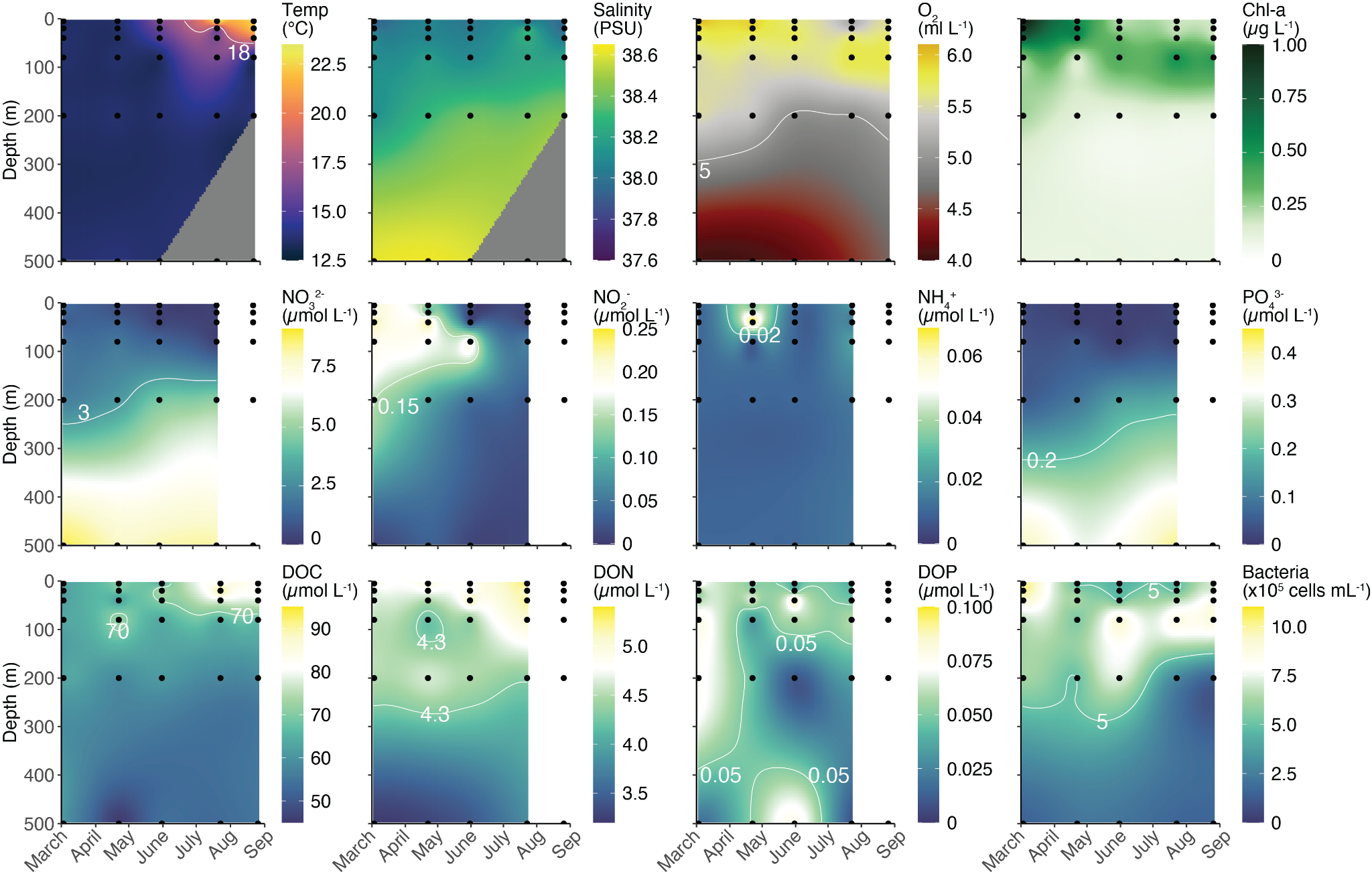
**

**Supplementary Figure 1 - Physicochemical environmental conditions at the Microbial Oceanography Laboratory Arago (MOLA) time series from spring to summer 2021.** Grey shading indicates that no samples were taken.


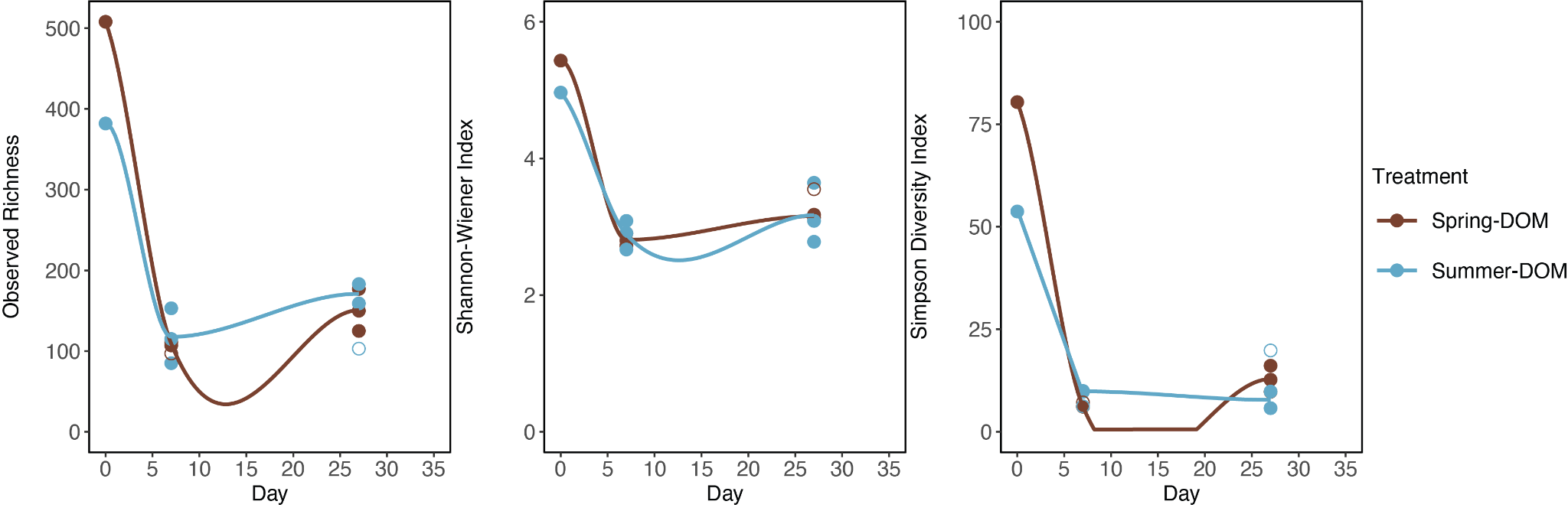


**Supplementary Figure 2 -** Experimental changes in alpha-diversity of prokaryotes composition.


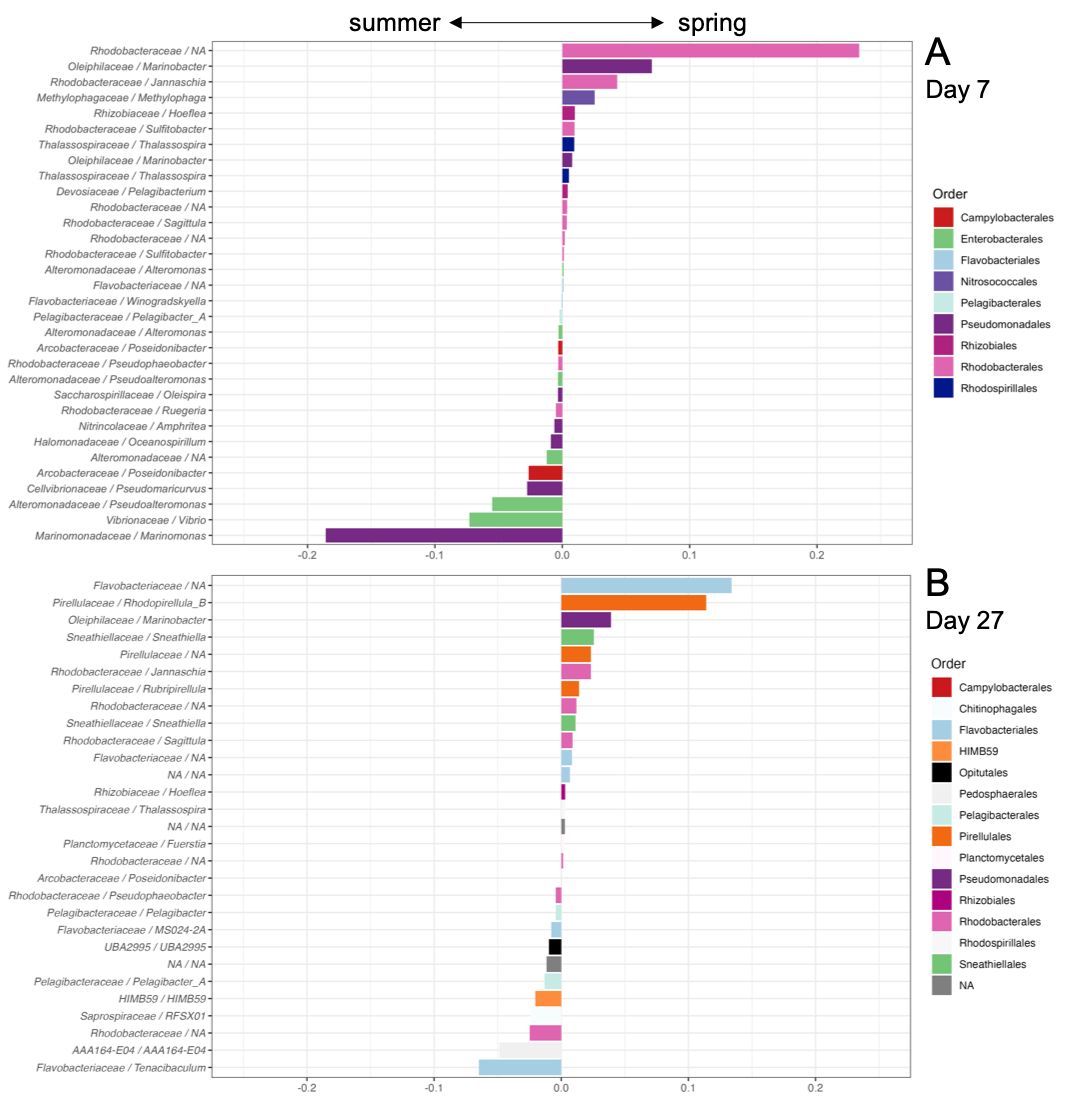


**Suppl. Figure 3.** Change in relative abundance of the more abundant ASV’s significantly (1 way ANOVA, p < 0.05) enriched in the spring-DOM treatment (right side of the panels) or in the summer-DOM treatment (left side of the panels) at day 7 (top panel) and day 27 (bottom panel). Colors denote the different prokaryote orders.


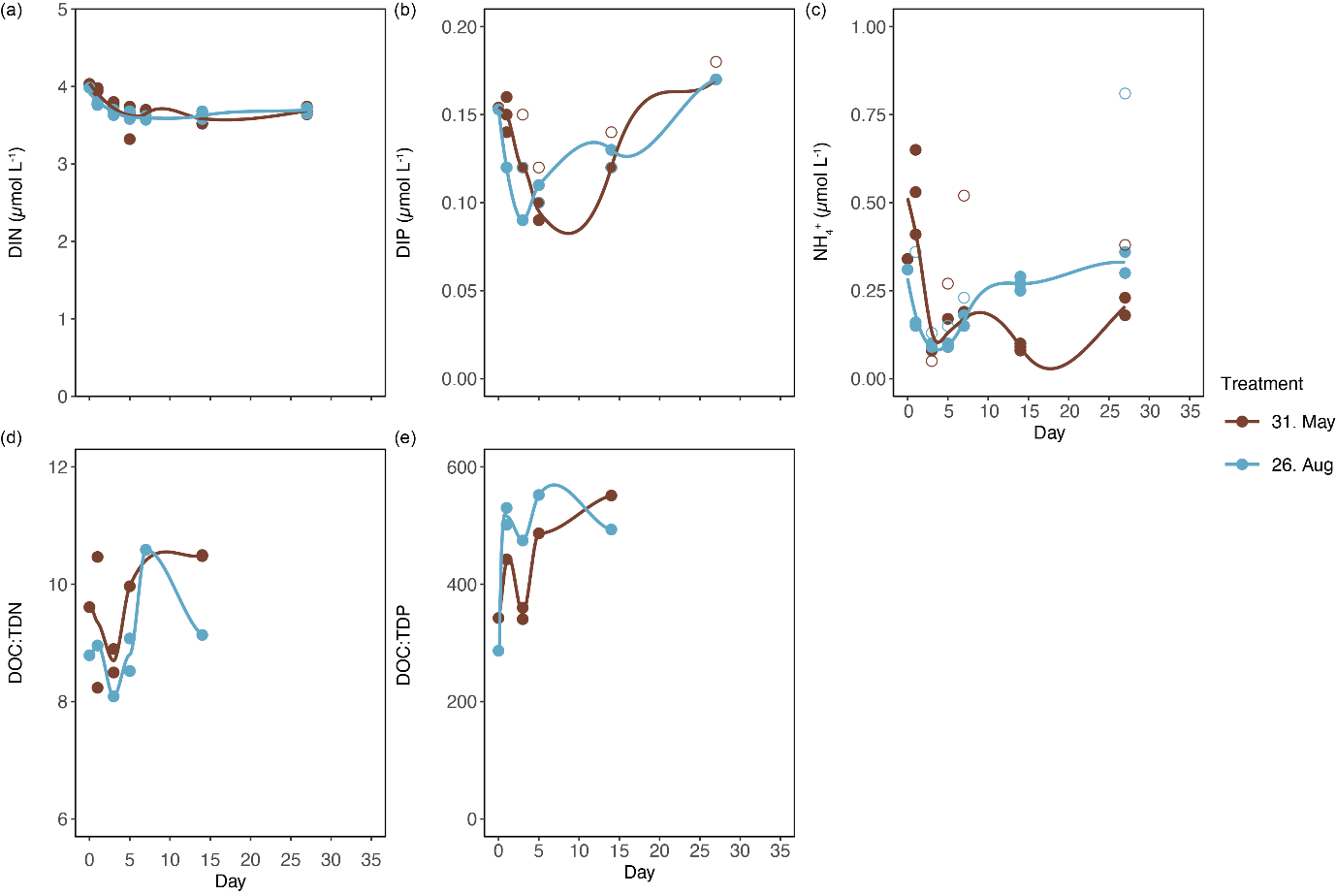


**Suppl. Figure 4 - Experimental changes in dissolved nutrients.** Changes in (a) dissolved inorganic nitrogen, (b) Dissolved inorganic phosphorus (c) Ammonium (d) dissolved organic carbon to total dissolved nitrogen ratios and (d) dissolved organic carbon to total dissolved phosphorus ratios

**
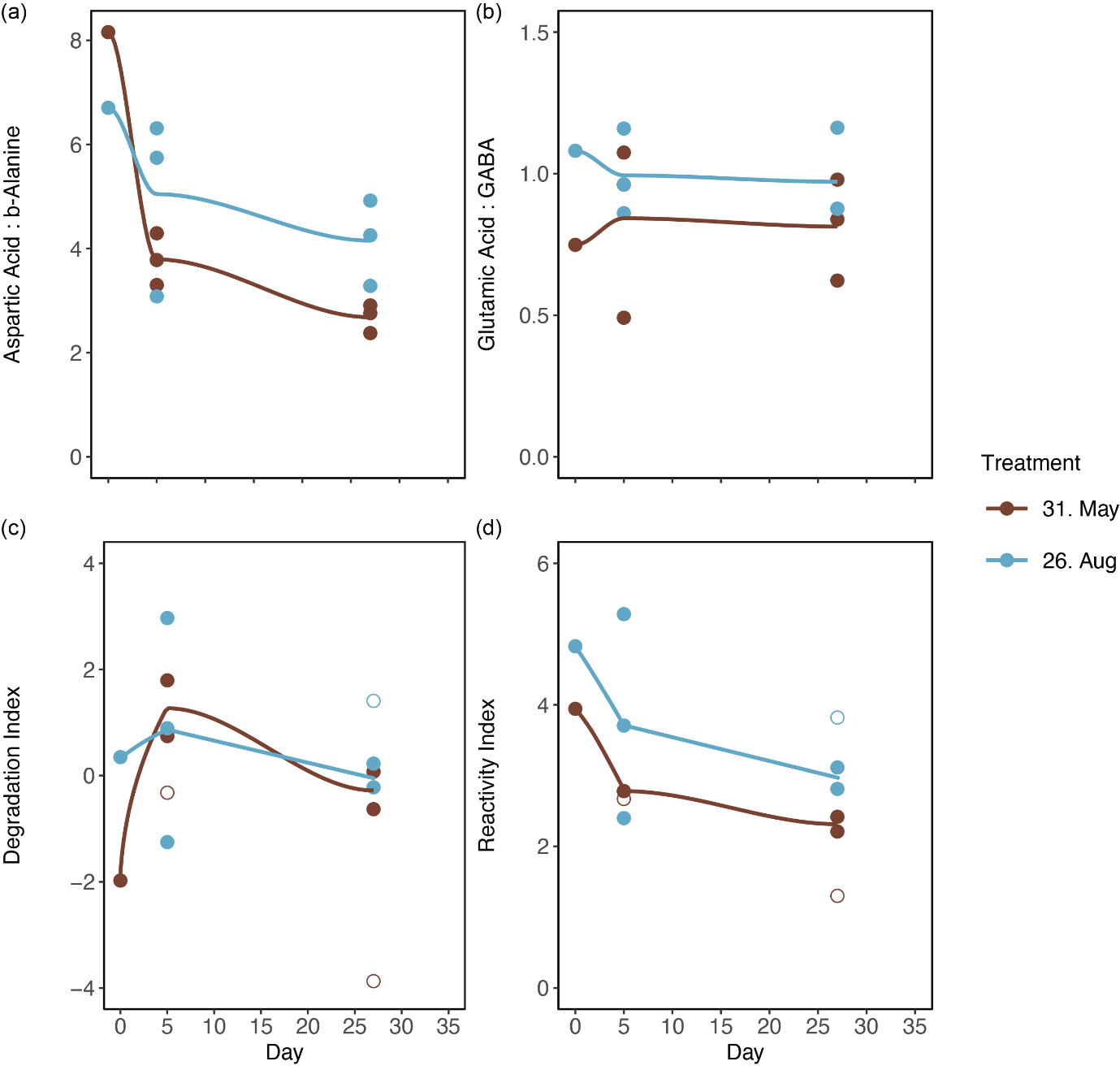
**

**Suppl. Figure 5. Ratios of dissolved hydrolyzable amino acids (DHAA) over time at the two different treatments.**

**
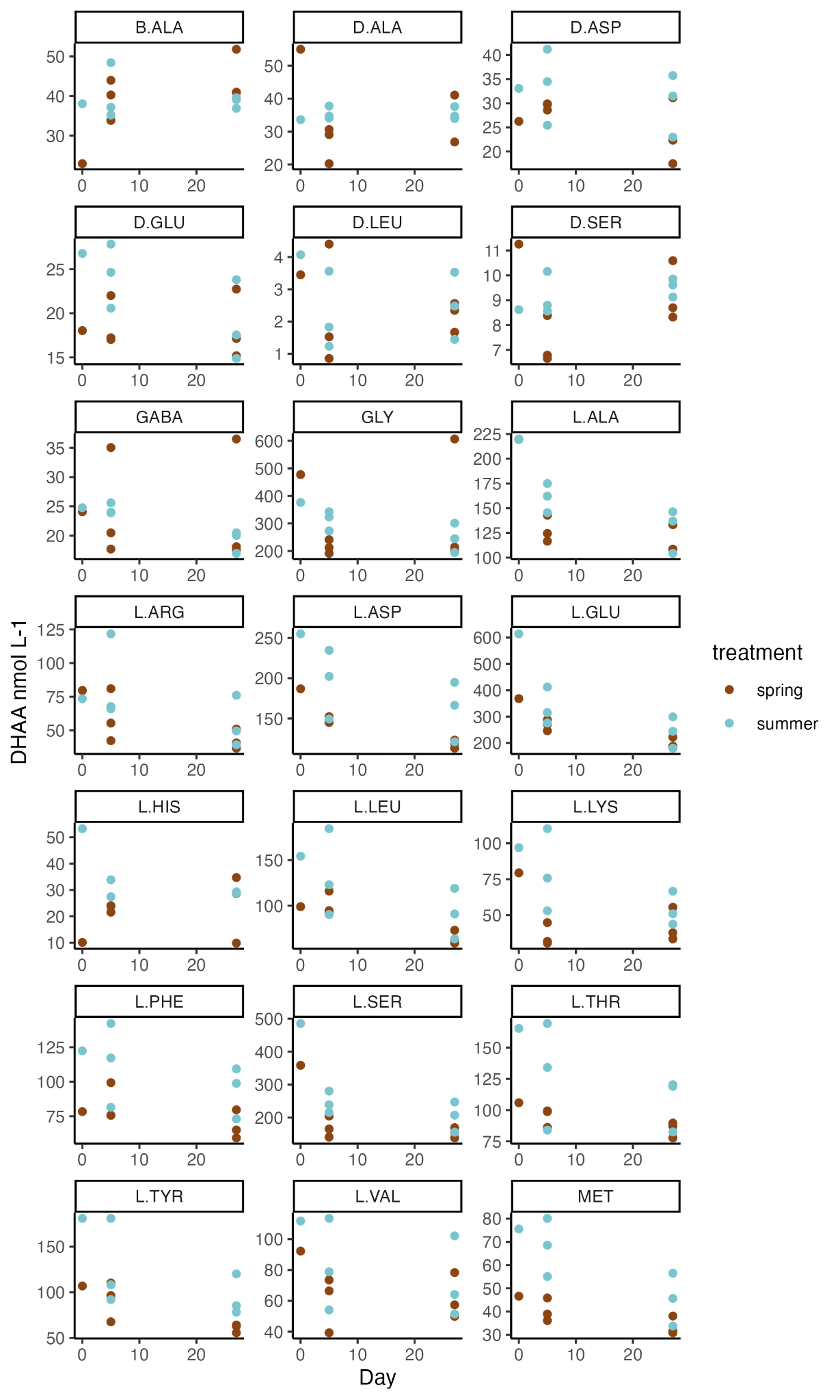
**

**Suppl. Figure 6. Concentration of individual L- and D- dissolved hydrolyzable amino acids (DHAA) over time at the two different treatments**

**Supplementary Table 1. Results of experiment at t0.** PA= Prokaryotic Abundance. PHP= Prokaryotic heterotrophic production. VA= Viral abundance. DIP= Dissolved inorganic phosphorus. DIN= Dissolved inorganic nitrogen. DOC= Dissolved organic carbon. DON= Dissolved organic nitrogen. DOP= Dissolved organic phosphorus. TDN= Total dissolved nitrogen. TDP= Total dissolved phosphorus. C:N:P: DOC:DON:DOP ratios. FDOM: Fluorescent dissolved organic matter. DHAA= Dissolved hydrolyzable amino acids.

| **Parameter at t0** | **Spring-DOM**  **Treatment** | **Summer-DOM**  **Treatment** |
| --- | --- | --- |
| PA (x10^5^ cells mL^-1^) | 0.49 | 0.65 |
| PHP (µgC L^-1^ d^-1^) | 8.33 | 11.65 |
| VA (x10^5^ particles mL^-1^) | 3.14 | 3.38 |
| NH4 (µmol L^-1^) | 0.34 | 0.31 |
| DIN (µmol L^-1^) | 4.03 | 3.98 |
| DIP (µmol L^-1^) | 0.15 | 0.15 |
| DOC (µmol L^-1^) | 83.5 | 82.5 |
| DON (µmol L^-1^) | 4.32 | 5.09 |
| DOP (µmol L^-1^) | 0.09 | 0.13 |
| TDN (µmol L^-1^) | 8.69 | 9.39 |
| TDP (µmol L^-1^) | 0.24 | 0.29 |
| C:N:P | 934:48:1 | 564:38:1 |
| FDOM Protein-like Peak T (x10^-3^ RU) | 185 | 153 |
| FDOM Humic-like Peak C (x10^-3^ RU) | 93.9 | 81.1 |
| FDOM Humic-like Peak M (x10^-3^ RU) | 116.4 | 93.9 |
| L-DHAA (nmol L-1) | 684.9 | 809.7 |
| D-DHAA (nmol L-1) | 32.8 | 28.4 |
